# Recursive evolution of spin-wave multiplets in magnonic crystals of antidot-lattice fractals

**DOI:** 10.1038/s41598-021-00417-0

**Published:** 2021-11-19

**Authors:** Gyuyoung Park, Jaehak Yang, Sang-Koog Kim

**Affiliations:** National Creative Research Initiative Center for Spin Dynamics and Spin-Wave Devices, Nanospinics Laboratory, Research Institute of Advanced Materials, Department of Materials Science and Engineering, Seoul National University, Seoul, 151-744 Republic of Korea

**Keywords:** Materials science, Condensed-matter physics, Ferromagnetism, Magnetic properties and materials

## Abstract

We explored spin-wave multiplets excited in a different type of magnonic crystal composed of ferromagnetic antidot-lattice fractals, by means of micromagnetic simulations with a periodic boundary condition. The modeling of antidot-lattice fractals was designed with a series of self-similar antidot-lattices in an integer Hausdorff dimension. As the iteration level increased, multiple splits of the edge and center modes of quantized spin-waves in the antidot-lattices were excited due to the fractals’ inhomogeneous and asymmetric internal magnetic fields. It was found that a recursive development (F_n_ = F_n−1_ + G_n−1_) of geometrical fractals gives rise to the same recursive evolution of spin-wave multiplets.

## Introduction

A recursive sequence is one of the most fundamental growth mechanisms in nature, interest in it having grown significantly for its potential quantum applications^1–3^. That is, a successive descendant of the nth generation is an aggregation of one or more preceding ascendants and their variations. More intriguing sequences are involved in fractal geometries in nature^4^. Those fractals are classified as statistical and random fractals^5^. Although the random fractals are relatively sporadic and weakly self-similar, the deterministic (exact) fractals have regularity and strong self-similarity. The recursive ordering has been discovered in the context of those two different fractal growths. Despite their close relationship, the recursive sequences in fractal growths have barely been studied in ordered spin systems.

Meanwhile, spatial periodicities of spin ordering in lattice crystals lead to the modifications of magnonic properties such as band structure^6–8^ and quantization^9–12^ of spin-waves. The antidot-lattice, a periodic array of many holes in a continuous film, has been a basic and promising two-dimensional (2D) magnonic crystal due to its scalability and good hysteric effect without superparamagnetic bottleneck^13^. The alteration of internal magnetic fields in the antidot-lattices results in several non-propagating eigenmodes even in forbidden bands. For example, edge modes, which resemble a “butterfly state”^10^, are localized around the boundary of each antidot, while center modes are extended along the channel in between the neighboring holes (antidots)^12,14^. Furthermore, standing spin-wave modes can be excited by different field conditions as well as in geometric confinements^15^. A variety of types of antidot-lattices have been employed that include bi-component (of different materials^16^ or different sizes of holes^17^), different Bravais type^18^, and defective lattices^19^. On the other hand, non-trivial magnonic dynamic behaviors were observed in aperiodic structures of antidots such as magnonic quasicrystals of Fibonacci structure^20–22^, Penrose and Ammann tilings^23^, and Sierpiński carpet^24–26^. In fact, Sierpiński fractals have led to unique phenomena in electronics^27,28^ and photonics^29,30^.

### Model system of antidot-lattice fractals

**Figure 1:**
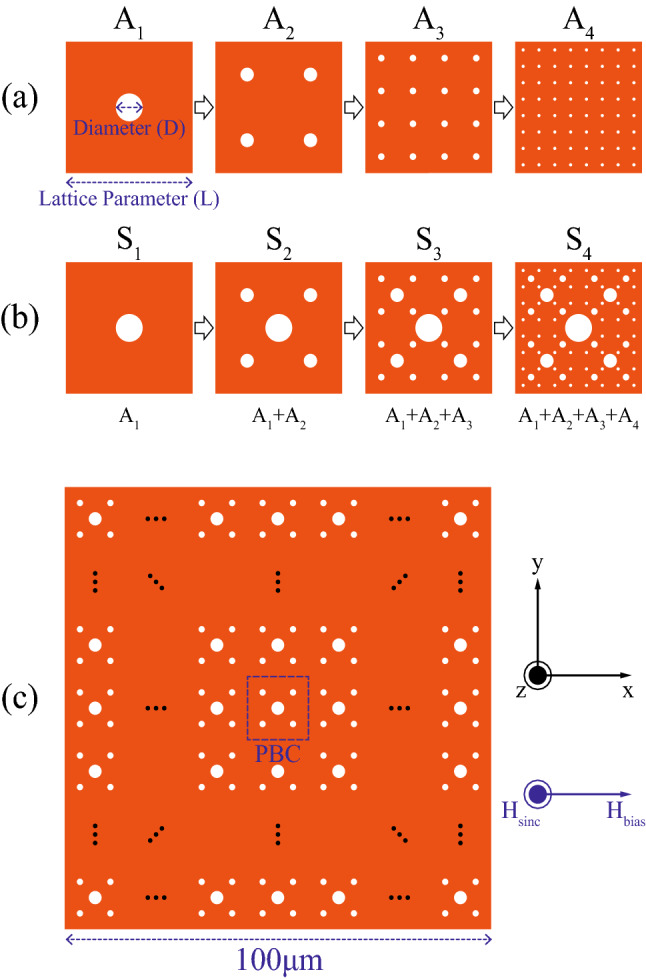
(**a**) Sequence of antidot-lattices with self-similar geometry. The capital letters of D and L correspond to the diameter of a hole and the size of a square Bravais lattice, respectively. (**b**) Evolution of antidot-lattice fractals. Each motif of S_n_ denotes the superposition of the individual lattices of A_1_, A_2_, … and A_n_. (**c**) Fractal magnonic crystal of 100 μm × 100 μm dimensions where periodic boundary condition was applied using the area marked by blue-dashed box (S_2_ as an example). For excitations of all possible spin-wave modes, dc-bias and sinc-function magnetic fields were applied in the + x direction and along the z-axis, respectively.

Here, we propose magnonic crystals composed of ferromagnetic antidot-lattice fractals, arranged similarly to one of Sierpiński aperiodic motifs, as studied by micromagnetic simulations along with a delicate analysis of multiplet spin-wave modes. The overlap of scaled antidot-lattices in a regular routine yields deterministic fractals, e.g., periodic structures with a local aperiodicity. This controllable non-statistical geometry provides a self-similarity in a part of the structure at every magnification, and can be designated according to the Hausdorff dimension of ${\mathrm{log}}_{\mathrm{S}}\mathrm{N}$, where S is the scale factor and N is the number of scaled objects^31^. That is, we used a series of scaled antidot-lattices (Fig. 1a) to construct antidot-lattice fractals deterministically with iterations, as illustrated in Fig. 1b. The antidot-lattices are self-similar in their geometric parameters of diameter D and lattice constant L. The D_n_ and L_n_ of the nth antidot-lattice (A_n_) are exactly half of those of A_n−1_. For example, A_2_ has ${\text{L}}_{2} = {\text{L}}_{1} /2$ and ${\text{D}}_{2} \,{ = }\,{\text{D}}_{1} /2$. Next, the nth fractal S_n_ is constructed by the superposition of A_1_ + A_2_ + ⋯ + A_n_ as follows: S_1_ = A_1_, S_2_ = A_1_ + A_2_, S_3_ = A_1_ + A_2_ + A_3_, and S_4_ = A_1_ + A_2_ + A_3_ + A_4_, as depicted in the series up to S_4_ (see Fig. 1b). In detail, A_1_ with D = D_1_ and L = L_1_ corresponds to the initiator (mother). A_1_ and A_2_ make up S_2_. Since the D_2_ and L_2_ of A_2_ are half those of A_1_, the number of antidots for A_2_ is increased by 4 times, and thus the Hausdorff dimension is log_2_4 = 2. Following A_n_ are scaled copies of previous A_n−1_ in the same manner. Due to the self-similarity of the fractals, a recursive sequence arises inside the geometry of the motifs: let F_n_ denote the geometrical sequence. F_n_ ($0 \le x \le L/2$) of each S_n_ is a summation of F_n−1_ ($L/4 \le x \le L/2$) and G_n−1_ ($0 \le x < L/4$). The appearance of F_n−1_ in S_n_ is a scaled recursion of F_n−1_ ($0 \le x \le L/2$) in S_n−1_. In S_n_, G_n−1_ is a variation of F_n−1_ and can be viewed as the A_1_ antidot superimposed onto F_n−1_.

Then, the 2D periodic lattice of magnonic crystals has a square Bravais symmetry, as shown in Fig. 1c.

## Results

### Recursive evolution of spin-wave multiplets

**Figure 2:**
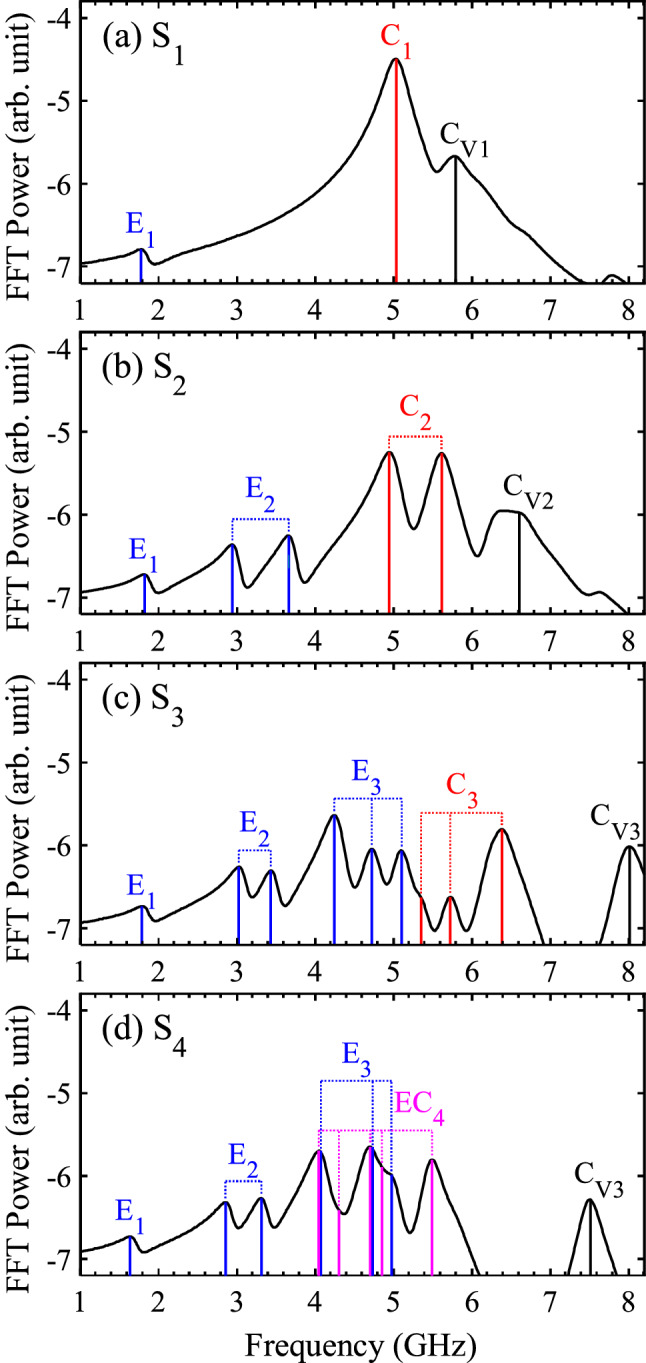
Modes’ spectra in magnonic crystals of antidot-lattice fractals, S_1_, S_2_, S_3_, and S_4_, with L_1_ = 1400 nm and D_1_ = 300 nm. E_n_ and C_n_ denote the edge and center mode of S_n_. A bias magnetic field of 30 mT was applied in the + x direction.

Figure 2 reveals that the spin-wave eigenmodes in the antidot-lattice fractals split into multiplets according to the recursive sequence (for better spectra, see also Supplementary Fig. S1). The first ordinary crystal denoted as S_1_ (= A_1_) exhibited three normal standing spin-wave modes as indexed by E_1_, C_1_, and C_V1_^12^. The very weak mode (E_1_) at 1.77 GHz corresponds to the edge mode, and the strongest mode (C_1_) at 5.02 GHz to the center mode. The two modes are periodically excited along the bias field direction. The last minor mode (C_V1_) at 5.80 GHz is a center-vertical mode (or a fundamental-localized mode) at the center between the neighboring antidots along the axis perpendicular to the bias field direction.

For S_2_, while keeping the E_1_ mode at a similar frequency, an additional doublet (E_2_) of the edge mode appeared, which originated from A_2._ The doublet (C_2_) of the center mode appeared as its substitution for the singlet C_1_ in S_1_ (see Fig. 2b). The higher mode (5.61 GHz) of the doublet C_2_ was hybridized with the C_V1_ mode (5.80 GHz) in S_1_. In a similar manner, for S_3_, an additional triplet (E_3_) came from A_3_, while the doublet C_2_ in S_2_ then became the triplet C_3_. The center-vertical mode (C_V2_) of A_2_ was hybridized with the highest mode (6.38 GHz) of C_3_. To sum up, by adding A_2_ and A_3_ to S_2_ and S_3_, respectively, each E_n_ mode was newly updated while the C_n_ mode substituted for C_n−1_. This happened because the edge mode is strongly localized at the boundary of each antidot while the center mode is extended through the channels between the neighboring holes in the antidot-lattices. Whenever the next scaled antidot-lattices were overlapped to the previous one, each boundary of antidot entity of A_n_ and A_n−1_ remained intact with each other, while the channels of A_n_ were impacted by the channels of A_n−1_.

In the case of A_4_, the edge mode and the center mode were hybridized into one mode, because the hole-to-hole distance was smaller than the previous antidot-lattices: the dipolar and exchange interactions were equally dominant at the narrow channel of A_4_. Therefore, for S_4_, the hybrid mode (E_4_ + C_4_ $\to$ EC_4_) appeared instead of the individual E_4_ and C_4_ modes. A total of five EC_4_ modes (pink-colored peaks) substituted for the C_3_ triplet.

The number of multiplets in the serial spectra followed the recursive sequence, F_n_ = F_n−1_ + G_n−1_. The two eigenmodes (E_n_ and C_n_) appeared as a singlet, doublet, triplet, and quintet for S_1_, S_2_, S_3_ and S_4_, respectively. Since there is only one zeroth (n = 0) standing spin-wave mode in unpatterned (continuous) thin film (i.e., S_0_), the number of the split modes corresponded to 1, 1, 2, 3, and 5 for n = 0, 1, 2, 3, and 4, respectively, for both E_n_ and C_n_. The difference sequence (G_n_) is 1, 1, and 2 for n = 1, 2, and 3, respectively.

**Figure 3:**
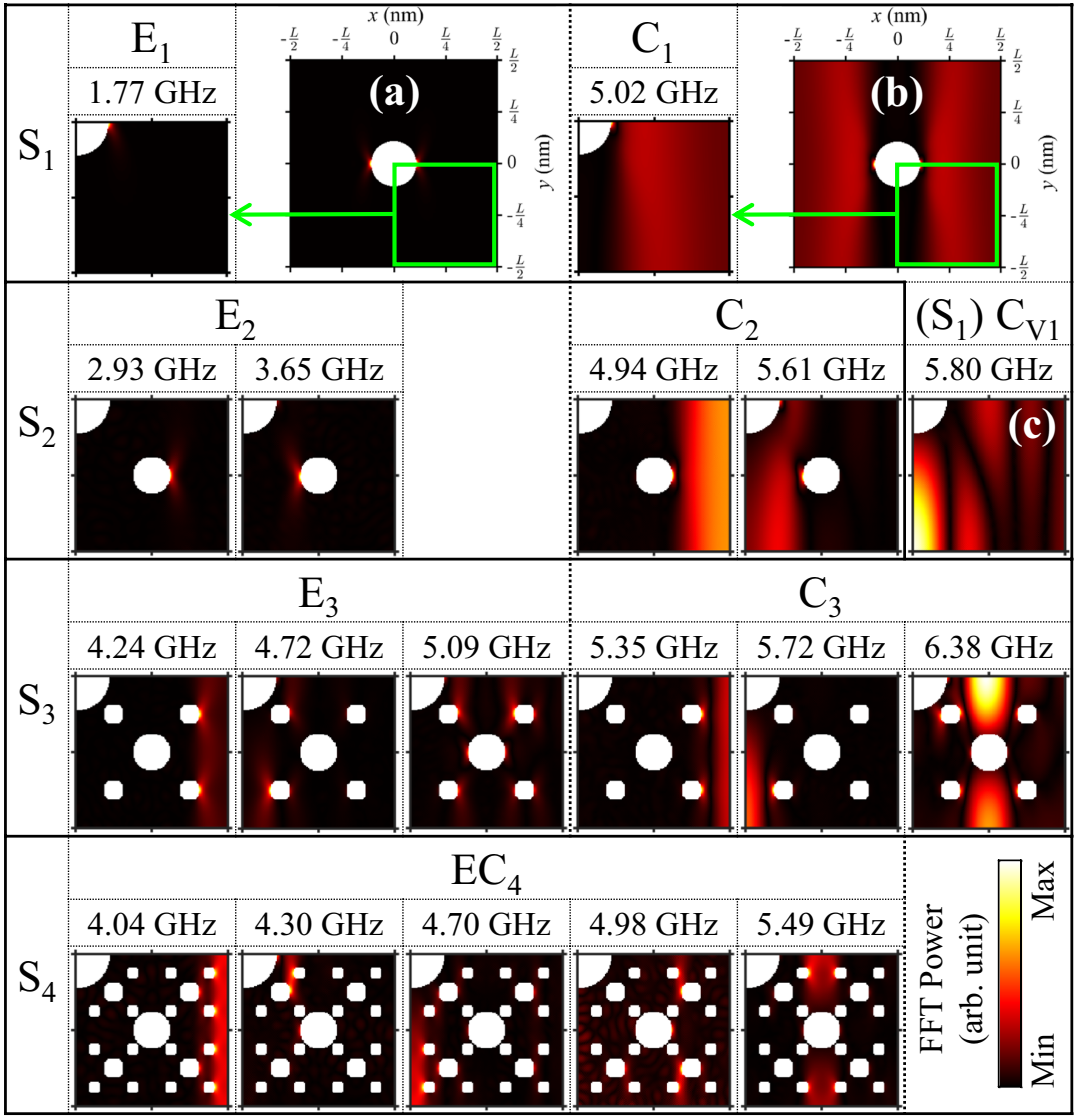
Spatial distribution of power of FFTs in bottom-right quarter area of motifs of S_1_, S_2_, S_3_ and S_4_ at indicated frequencies of specific modes.

In order to identify all of the excited modes represented by the FFT power-vs.-frequency spectra shown in Fig. 2, we performed FFTs on every single unit cell (or mesh) at the indicated resonance frequencies of the modes. Figure 3 shows the spatial distributions of FFT power in the bottom-right quarter of each motif ($0 \le x \le L/2$, $- L/2 \le y \le 0$) for each resonance frequency of the excited modes (for the corresponding phase profiles, see Supplementary Fig. S2). For S_1_, the major modes of E_1_ and C_1_ were visualized at 1.77 and 5.02 GHz, respectively. The edge mode was excited at the edge (or end) of the antidot (Fig. 3a), while the center mode was excited at the center (or channel) of the neighboring antidots (Fig. 3b). The higher mode (5.61 GHz) of the C_2_ doublet, was vertically localized between the neighboring holes of A_1_ in which C_V1_ was also localized in S_1_ (Fig. 3c). This explains why the higher mode of C_2_ was hybridized with C_V1_, as mentioned earlier. On the other hand, the lower (4.94 GHz) one of C_2_ remained extended along the channel between S_2_. The doublets of both E_2_ and C_2_ are antiphase with each other in temporal oscillation; S_2_ can be considered to be a two-dimensional nano-oscillator. For S_3_, the highest C_3_ at 6.38 GHz was fully localized in between the A_2_ antidots along the y-axis: it was hybridized with C_V2_ due to their shared localization area. The lowest C_3_ (5.35 GHz) remained extended along the channel between S_3_. Similarly, the lowest EC_4_ (4.04 GHz) for S_4_ was the only extended mode, while the others were localized in different local regions as noted by the red color. Since the distance between the antidots of A_3_ and A_4_ are close, some localized modes (4.30 GHz and 4.98 GHz) were excited at antidots of A_3_ together with certain antidots of A_4_. On the other hand, some of the E_3_ modes (4.04 GHz and 4.70 GHz) of S_4_ were excited at the same frequencies as those of the EC_4_ modes. Those E_3_ and EC_4_ modes become separated when the intensity of the bias field increased (see Supplementary Fig. S3).

The gap between the split modes narrowed down and finally merged into a singlet as the inhomogeneity of the magnetic energy decreased. In the other direction, the gap became wide and the corresponding different modes crossed over each other (showed conversion) as the inhomogeneity of the magnetic energy increased.

**Figure 4:**
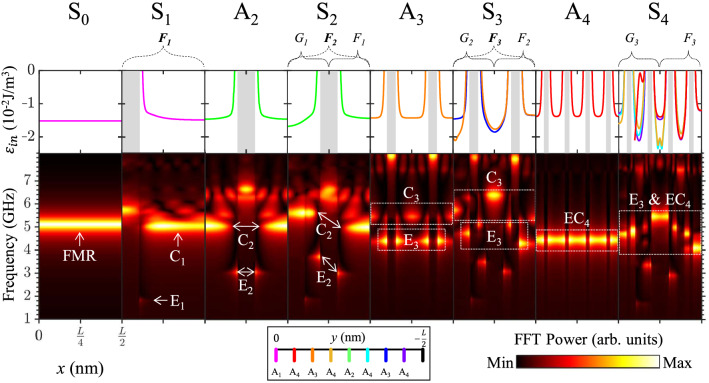
(Upper row) Spatial distributions of total magnetic energy density for thin film (S_0_) and magnonic crystals of A_n_ and S_n_. The energy densities were plotted by cross-section along the y-axis where corresponding antidots were located. Each color of the plot matches with the index of the y-slice (black box) at the bottom of the figure. The gray-colored regions inside the plots denote the locations of the antidots. (Bottom row) Contour plots of FFT power on frequency and x position.

Similar to the recursive sequence in the geometrical fractals, we also found the recursive sequence in the evolution of the eigenmodes’ spatial profiles, as shown in Fig. 4. In detail, for the E_n_ modes, let E_1_ in S_1_ be F_1_. The two E_2_ modes in S_2_ are F_2_. The right part (L/4 $\leq$ x $\le$ L/2) of the E_2_ (2.93 GHz) profile is the same as the E_1_ profile in S_1_. E_2_ (2.93 GHz) is F_1_ in S_2_. The left part (0 $\le$ x $<$ L/4) of the E_2_ (3.65 GHz) profile is a variation of the E_2_ (2.93 GHz) profile. E_2_ (3.65 GHz) is G_1_ in S_2_. The three E_3_ modes in S_3_ are F_3_. The right parts of the E_3_ (4.24 GHz and 5.09 GHz) profiles are similar to the E_2_ profiles in S_2_. The two E_3_ modes are F_2_ in S_3_. The left part of the E_3_ (4.72 GHz) profile is a variation of the E_3_ (4.24 GHz) profile. E_3_ (4.72 GHz) is G_2_ in S_3_. The five EC_4_ modes in S_4_ are F_4_. The right parts of the EC_4_ (4.04 GHz, 4.98 GHz, and 5.49 GHz) profiles are similar to the E_3_ profiles in S_3_. The three EC_4_ modes are F_3_ in S_4_. The left parts of the EC_4_ (4.30 GHz and 4.70 GHz) profiles are variations of the EC_4_ (4.98 GHz and 4.04 GHz, respectively) profiles. The two EC_4_ (4.30 GHz and 4.70 GHz) modes are G_3_ in S_4_. In the same way, let C_1_ in S_1_ be F_1_. The two C_2_ modes in S_2_ are F_2_. The right part of the C_2_ (4.94 GHz) profile is the same as the C_1_ profile in S_1_. C_2_ (4.94 GHz) is F_1_ in S_2_. The left part of the C_2_ (5.61 GHz) profile is a variation of the C_2_ (4.94 GHz) profile. C_2_ (5.61 GHz) is G_1_ in S_2_. The three C_3_ modes in S_3_ are F_3_. The right parts of the C_3_ (5.35 GHz and 6.38 GHz) profiles are the same as the C_2_ profiles in S_2_. C_3_ (5.35 GHz and 6.38 GHz) are F_2_ in S_3_. The left part of the C_3_ (5.72 GHz) profile is a variation of the C_3_ (5.35 GHz) profile. C_3_ (5.72 GHz) is G_2_ in S_3_. The EC_4_ modes are considered in the same way as mentioned above.

### Origin of recursive evolution

Next, in order to identify the splits of the spin-waves excited in S_n_ with respect to A_n_, we plotted the contours of FFT power for the frequency and the longitudinal x-direction, as shown in the bottom row of Fig. 4. In the upper row of Fig. 4, we also plotted the spatial distributions of the total magnetic energy density (${\mathcal{E}}_{\mathrm{tot}}$), as expressed by

1 $$\varepsilon_{tot} = - \frac{{\mu_{0} }}{{2V_{mesh} }}\int {\textbf{\textit{M}} \cdot ({\textbf{\textit {H}}_{\textbf{\textit {Zeem}}}} + {\textbf{\textit {H}}_{\textbf{\textit {demag}}}})dV_{mesh} + A_{exch} \int {\left| {{\varvec \nabla} \textbf{\textit{M}}} \right|} }^{2} dV_{mesh} ,$$

where **H**_**Zeem**_ and **H**_**demag**_ are the Zeeman and demagnetization fields, respectively, **M** is the magnetization, A_ex_ is the exchange constant, and V_mesh_ is the volume of the mesh. The gray-colored regions depict the locations of the antidots inside each motif. The color of each plot matches with the y-slice index (the black box) at the bottom of Fig. 4. The FFT powers along the x distance ($0 \le x \le L/2$) agree well with the spatial distributions of the total energy density in terms of the x position. For example, the FMR mode was excited in the thin film (denoted as S_0_) at 5.10 GHz, as indicated by the homogeneous total energy distribution. The recursive sequence (F_n_) marked at the top of Fig. 4 denotes the evolution of both the total magnetic energy and the frequency of the eigenmodes. The right region ($L/4 \le x \le L/2$) of S_n+1_ is F_n_. The appearance of F_n_ in S_n+1_ is similar to F_n_ in S_n_. The left region ($0 \le x < L/4$) of S_n+1_ is G_n_, which is a variation of F_n_ in S_n+1_.

In S_1_, at a similar frequency to that of the FMR mode, the C_1_ mode was excited at 5.02 GHz in the region of $D/2 < x \le L/2$. To be specific, the FFT power spatially informed that the C_1_ mode started to be excited at the end of the E_1_ mode (1.77 GHz) in terms of the x position. This profile well matches the total energy distribution of S_1_ (= A_1_). The magnetic energy variation inside the magnonic crystal corresponds to the localization of the quantized spin-wave modes. The mode arrangement of A_2_ was similar to that of A_1_. The C_2_ mode of A_2_ was excited at 5.03 GHz, whereas the E_2_ mode was excited at 3.00 GHz. With regard to S_2_, the E_2_ and C_2_ modes were split into doublets. Due to the existence of the A_1_ antidot on the left side of the A_2_ antidot, the energy profile on the left side is different from the right side of the A_2_ antidot. The C_V1_ mode and the shifted C_2_ mode were hybridized together, as shown in Fig. 3. In S_3_, the magnetic energy profile inside the motif was divided into three distinct regions. Therefore, the E_3_ mode at 4.42 GHz in A_3_ became split into three modes at 4.24, 4.72, and 5.09 GHz in S_3_. Similarly, the C_3_ mode at 5.50 GHz in A_3_ split into triplets (5.35, 5.72, and 6.38 GHz) in S_3_. In A_4_, the EC_4_ mode was excited at 4.45 GHz, and the excitation profile showed that the edge and center modes had been hybridized into a single mode. Then, for S_4_, the EC_4_ mode was split into 5 modes (quintets). Since the E_3_ mode of A_3_ and the EC_4_ mode of A_4_ were excited at almost an equal frequency, a total of 8 split modes (the E_3_ triplets plus the EC_4_ quintets) in S_4_ were mixed up. The total energy distribution of S_4_ is complicated compared with those of the previous motifs, because of the complex arrangement of antidots inside S_4_.

The self-similarity of the fractal motifs introduces aperiodic arrangements of antidots in recursive order. In S_n_, the total magnetic energy (most dominantly demagnetization energy) of the nth antidot array (A_n_) became aperiodic since the previous antidots modulated the magnetization configurations around A_n_ antidots. The aperiodic energy variation inside the antidot-lattice fractals gives rise to the multiplets of the spin-wave eigenmodes under the recursive evolution. The energy aperiodicity can be reduced according to the geometric parameters or the externally applied magnetic fields in order to make the magnons’ multiplets degenerate. As the dot-to-dot distance increased, the extent of aperiodicity decreased, and then the magnons’ modes became degenerated (see Supplementary Fig. S5). In the same way, as the strength of the external magnetic field increased, the split modes were reunited (see Supplementary Fig. S3).

### Origin of spin-wave multiplets

Finally, in order to examine the difference of the magnonic excitations between the fractal and non-fractal structures, we conducted the same simulation for the non-fractal, 2D type of NaCl lattice where two different radius holes are arranged alternately. This type of antidot-lattice has been studied under different terminologies, either composite-antidot array^32,33^ or bi-component antidot-lattice^16,17^. To avoid confusion, the term ‘2D NaCl type’ is employed to describe the antidot-lattice with alternating different diameters. The two antidot sublattices of A_1_ and A_2_ compose S_2_ as well as 2D NaCl type. In S_2_, they satisfy the initiator-generator relationship with ${\text{L}}_{2} = {\text{L}}_{1} /2$ and ${\text{D}}_{2} \,{ = }\,{\text{D}}_{1} /2$. In 2D NaCl type geometry, both antidots exist in the 1:1 ratio, since ${\text{L}}_{2} = {\text{L}}_{1}$ but ${\text{D}}_{2} \,{ = }\,{\text{D}}_{1} /2$. The location of the A_1_ antidot is asymmetric to that of the A_2_ antidot in the S_2_ motif, while it is symmetric in the 2D NaCl type motif. In both structures, antidots of L_1_ (L) = 1400 nm and D_1_ (D) = 300 nm were used, while a 30 mT strength of magnetic field was applied in the + x-direction.

**Figure 5:**
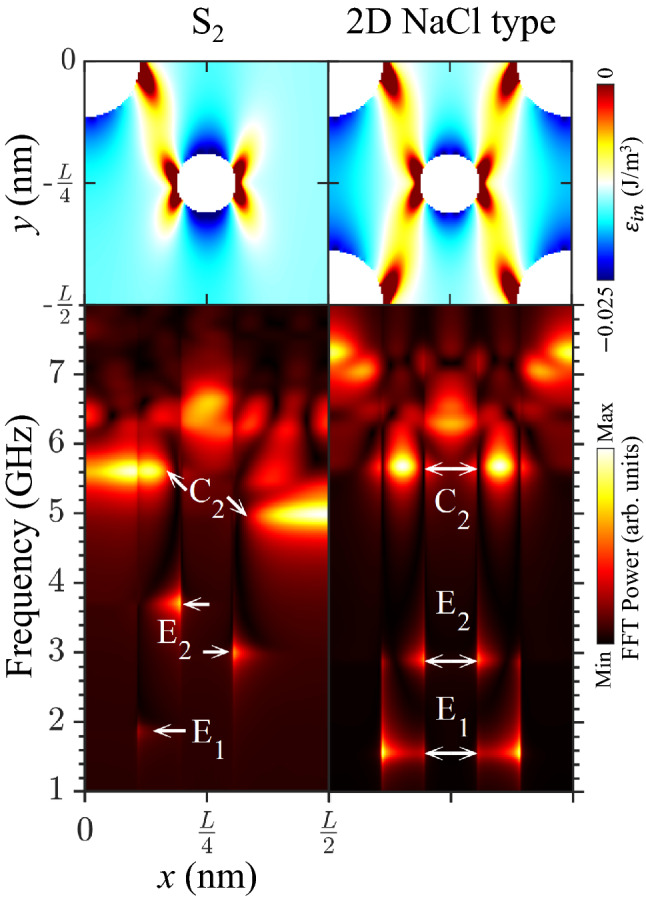
Comparison between S_2_ fractal and 2D NaCl type (non-fractal) magnonic crystals: (Upper row) spatial distribution of total magnetic energy density in bottom-right quarter area of given motifs. (Bottom row) Spatial distribution of frequency spectra of FFT power in x-direction. Doublets of E_2_ and C_2_ appeared only at the S_2_ fractal.

Figure 5 shows the FFT power versus frequency and the x position ($0 \le x \le L/2$) along with the total energy (${\upvarepsilon }_{\mathrm{tot}}$) density distribution. In the range of f = 1 ~ 6 GHz, the E_1_, E_2_ and C_2_ modes appeared noticeably in both S_2_ and 2D NaCl type. Both of the E_1_ modes were independent singlets derived from the A_1_ antidots in both patterns. The only difference was that the E_1_ mode in S_2_ was excited at a slightly higher frequency, since the total magnetic field near the ends of the A_1_ antidots in S_2_ was higher than that of 2D NaCl type. The E_2_ and C_2_ modes appeared as doublets only in S_2_, whereas those modes were typical singlets in 2D NaCl type. In a comparison of the energy distributions between the two structures, the above difference resulted from the asymmetry of the internal energy about $x = L/4$. Unlike S_2_, the non-fractal 2D NaCl type has the same energy distribution at both sides of the A_2_ hole; i.e., it shows a mirror symmetry about $x = L/4$. For S_2_, the asymmetry of the total magnetic energy inside the fractal magnonic crystal is the origin of the spin-wave multiplets.

## Discussion

The proposition of novel magnonic crystals composed of antidot-lattice fractals enlarges a basic understanding of quantized spin-wave modes. The fractals of 2-Hausdorff dimensions were constructed by the superposition of self-similar antidot-lattices. Local asymmetries inside the aperiodic magnonic motifs result in the split of the spin-wave eigenmodes: the edge mode, the center mode and the center-vertical mode (see Supplementary Fig. S5b,c). Due to the recursive sequence from the geometrical fractal growth, the local asymmetries inside the antidot-lattice fractals split the spin-waves into multiplets in the frequency spectra, showing the same recursive development. The split modes were finely localized into their own characteristic regions enabling selective excitation of the local area inside the magnonic crystals. Some of those split modes were reunited (or even duplicated) by the variations of the strength and direction of applied bias magnetic fields (see Supplementary Figs. S3 and S4, respectively). The reunion and the crossover among those finely divided modes would make the most of an active control with the bias magnetic field and the crystal geometry design (see Supplementary Fig. S5).

The proliferous standing spin-wave modes with fine localizations would be good candidates for magnonic devices that require diminutive excitation in a certain area of 2D nano-oscillators, memory devices, and sensors.

## Methods

### Micromagnetic simulation procedure

In the present simulations, we used an open-source software, MuMax3^34^, which incorporates the Landau-Lifshitz-Gilbert equation^35,36^ along with GPU acceleration to solve the dynamic motions of individual magnetizations in given magnonic crystals, for example, as shown in Fig. 1c. There, the motif, the S_2_ fractal marked by a dashed square box, was extended to sufficiently large dimensions (100 μm × 100 μm × 10 nm) with a periodic boundary condition in order to avoid the distortions of the static and dynamic magnetizations at the discontinuous boundaries of its finite dimensions. The sizes of unit cells in the simulations were set up to 5 nm × 5 nm × 10 nm. The material parameters used for Permalloy (Py: Ni_80_Fe_20_) were as follows: gyromagnetic ratio $\upgamma$ = 2.211 × 10^5^ [m/A s], saturation magnetization M_s_ = 8.6 × 10^5^ [A/m], exchange stiffness A_ex_ = 1.3 × 10^–11^ [J/m], damping constant $\mathrm{\alpha }$ = 0.01, and zero magnetic anisotropy constant, K_1_ = K_2_ = 0 [J/m^3^].

In order to excite spin-wave modes in the given magnonic crystals, we used a sinc (sine-cardinal) field as expressed by h(t) = h_0_sin[2πf_0_(t − t_0_)]/[2πf_0_(t − t_0_)] with $\mu_{0} {\text{h}}_{0}$ = 1 mT, ${\text{f}}_{0}$ = 20 GHz, t_0_ = 1 ns, and t = 100 ns. This pumping field was applied along the film normal under a dc bias field of $\mu_{0} {\text{H}}_{{{\text{bias}}}}$ = 30 mT applied in the + x direction on the film plane (The eigenmodes of antidot-lattice fractals were stabilized at magnetic fields of greater strength than 20 mT; see Supplementary Fig. S3). The temporal oscillations of local magnetizations at each cell were transformed into the frequency domain via Fast Fourier Transforms (FFTs).

## Supplementary Information

Supplementary Information.

Supplementary Movies.
