# Supplementary material for: Recursive evolution of spin-wave multiplets in magnonic crystals of antidot-lattice fractals
Source: Sci Rep. 2021 Nov 19;11:22604. doi: 10.1038/s41598-021-00417-0 (PMC8604906; doi:10.1038/s41598-021-00417-0)
Supplement: Supplementary file 1 — Supplementary Information. [file 41598_2021_417_MOESM1_ESM.docx]

**[SUPPLEMENTARY]**

**Recursive evolution of spin-wave multiplets**

**in magnonic crystals of antidot-lattice fractals**

Gyuyoung Park, Jaehak Yang, and Sang-Koog Kim^a)^

*National Creative Research Initiative Center for Spin Dynamics and Spin-Wave Devices, Nanospinics Laboratory, Research Institute of Advanced Materials, Department of Materials Science and Engineering, Seoul National University, Seoul 151-744, Republic of Korea*

^a)^ Correspondence and requests for materials should be addressed to S.-K. K (sangkoog@snu.ac.kr).


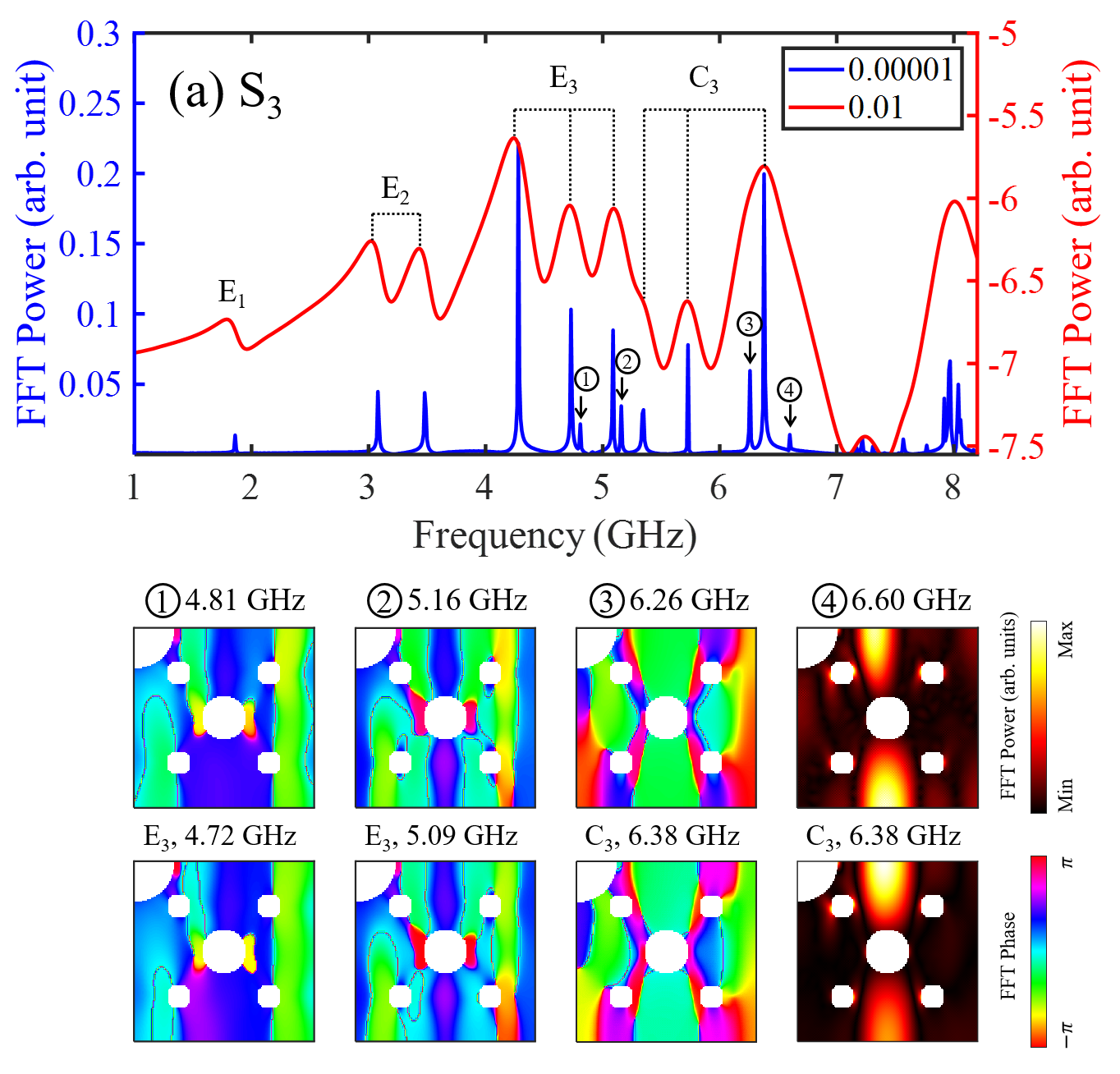


FIG. S1(a). Comparison of modes spectra for the *S*_3_ fractal obtained with $\alpha$ = $1\times{10}^{-5}$ (blue) and $\alpha$ = 0.01 (red). Much sharper and well separated peaks were found for $\alpha$ = $1\times{10}^{-5}$. The spatial distributions of the power and phase profiles are displayed for specific modes indexed by ①, ②, ③, and ④ along with the corresponding resonance frequencies.


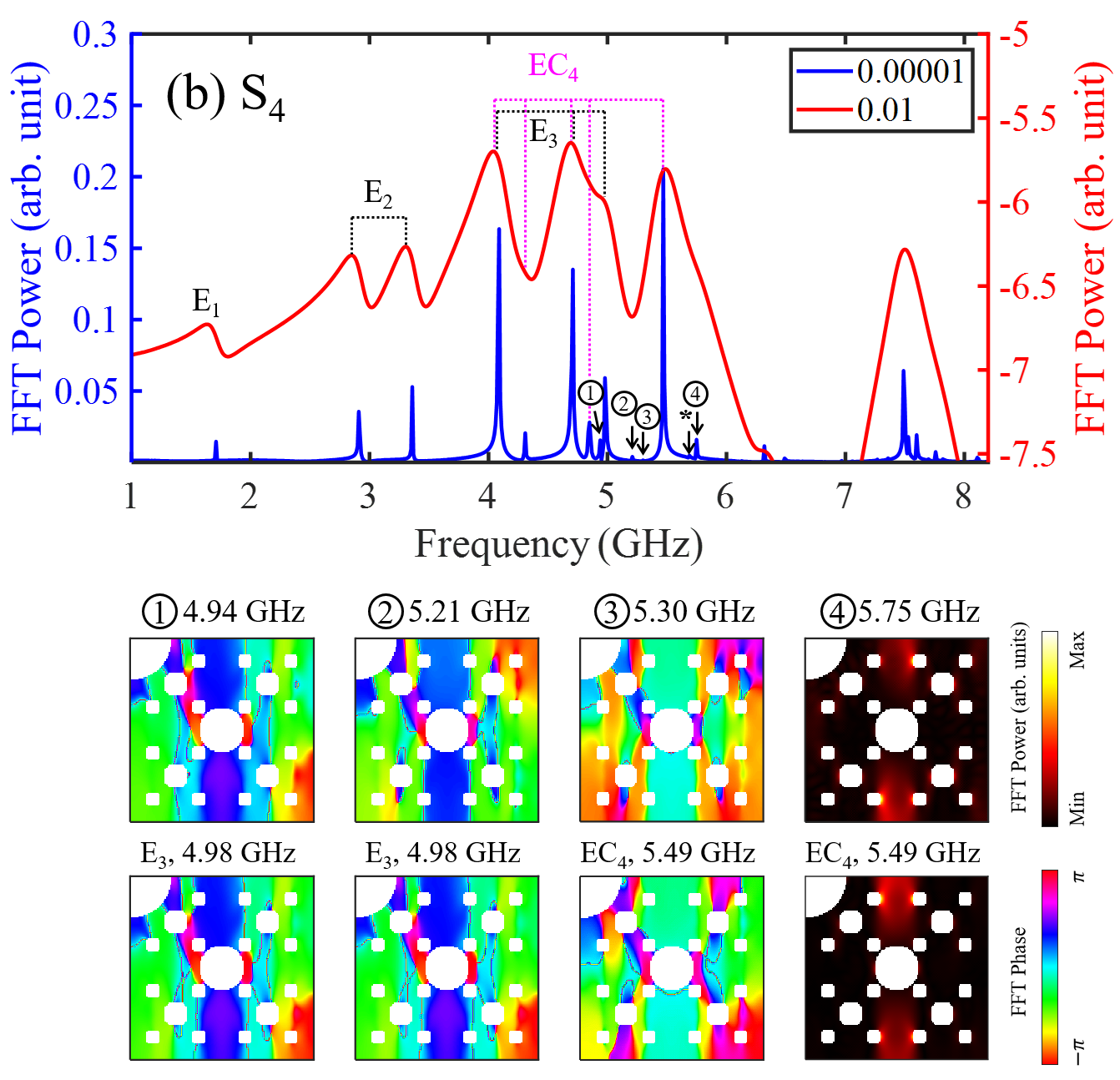


FIG. S1(b). Comparison of modes spectra for the *S*_4_ fractal obtained with $\alpha$ = $1\times{10}^{-5}$ (blue) and $\alpha$ = 0.01 (red). Much sharper and well separated peaks were found for $\alpha$ = $1\times{10}^{-5}$. The spatial distributions of the power and phase profiles are displayed for specific modes indexed by ①, ②, ③, and ④ along with the corresponding resonance frequencies.


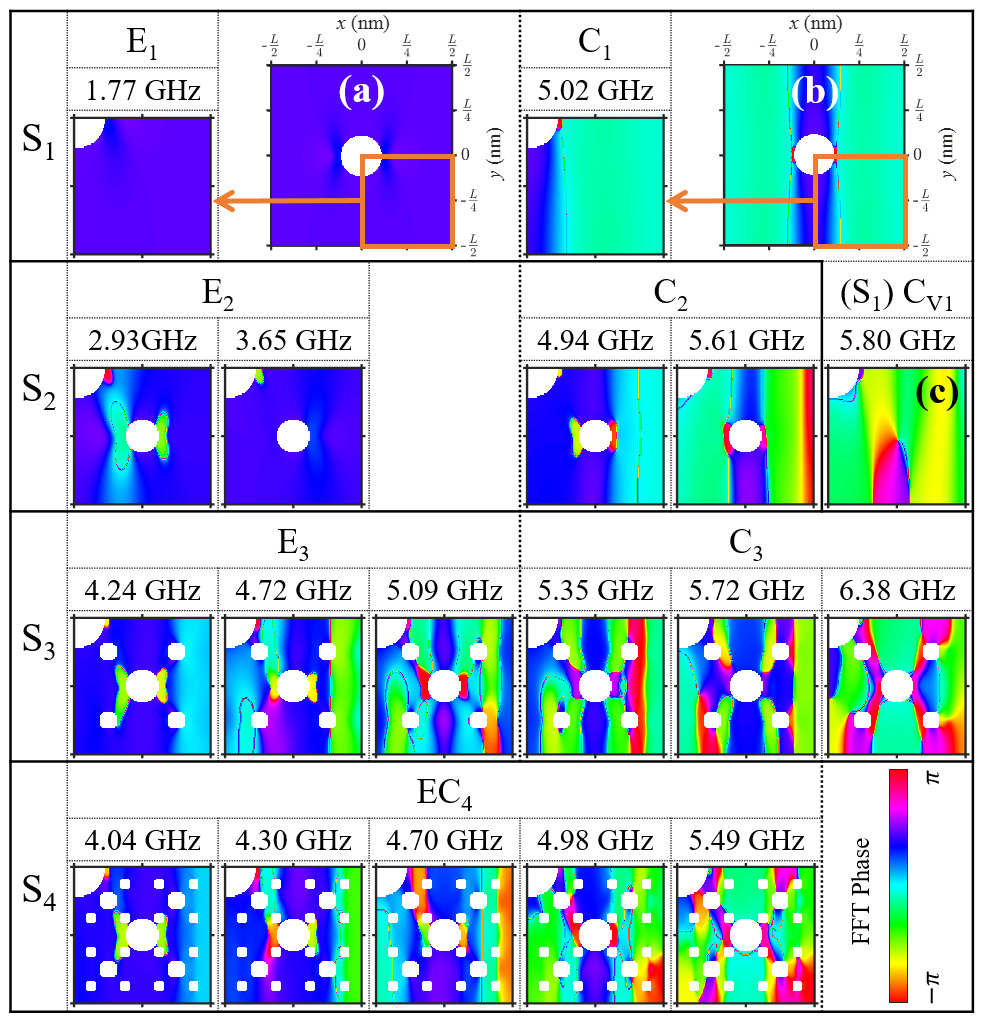


Fig. S2. Spatial distributions of the corresponding phases of the power profiles shown in Fig. 3 of the main manuscript.


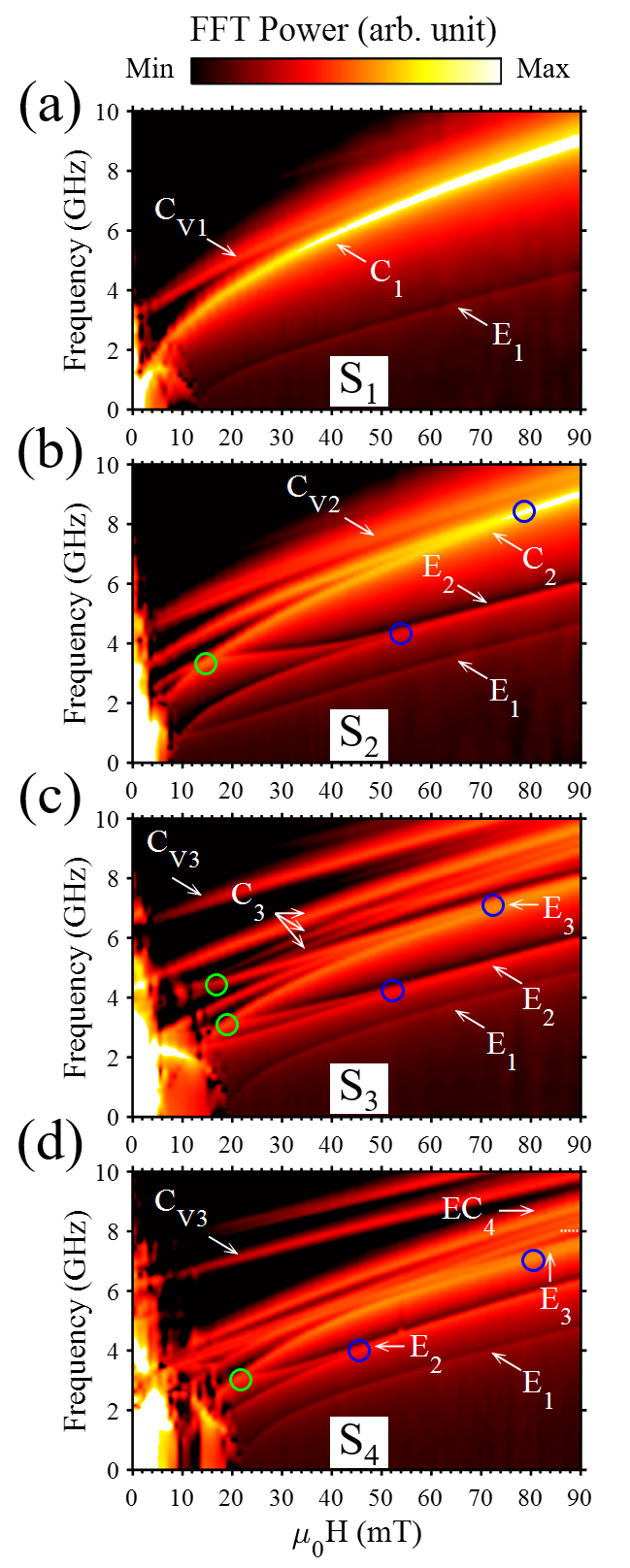


FIG. S3. Spin-wave modes excited in antidot-lattice fractals (*L* = 1400 nm and *D* = 300 nm) as a function of the strengths of bias magnetic fields applied in the +*x*-direction. The field strength was varied by every 2 mT in a range of 0 ~ 90 mT. The split modes were reunited in high-frequency regions (blue circles). Two different modes (e.g. higher E_2_ and lower C_2_ in S_2_) crossed over in low-frequency regions (green circles).


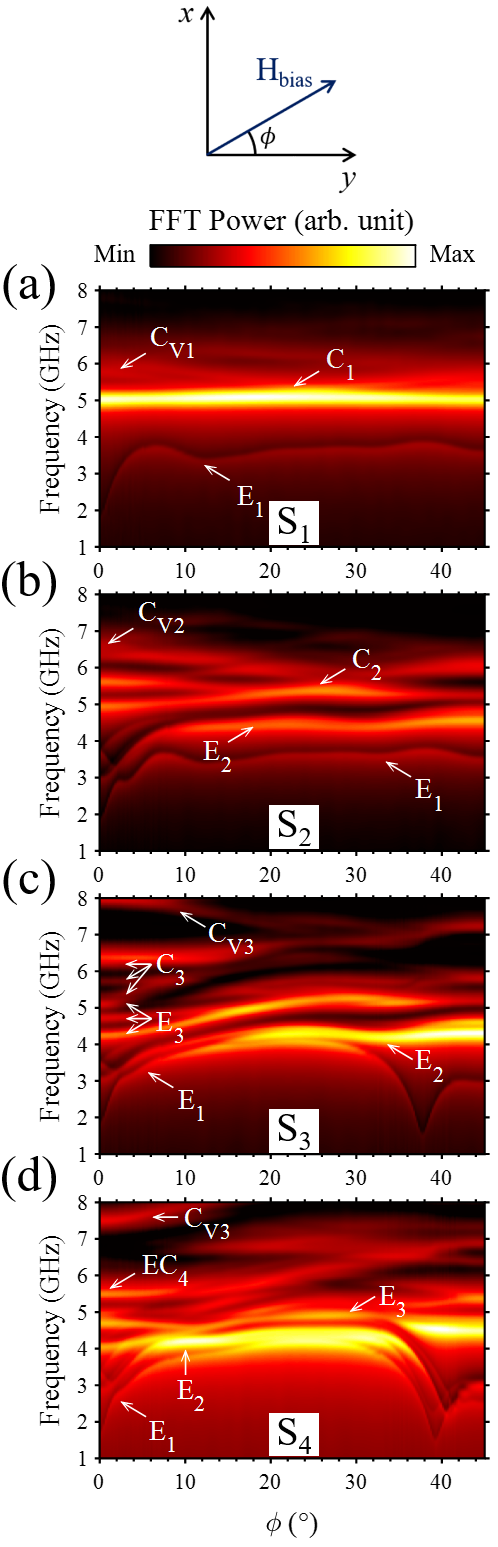


FIG. S4. Modes’ spectra as a function of $\phi$, the angle between *H* and the +*x*-axis, where $\phi$ was varied at every 0.1$^{\circ}$ in a range of 0$^{\circ}$ ~ 45$^{\circ}$.


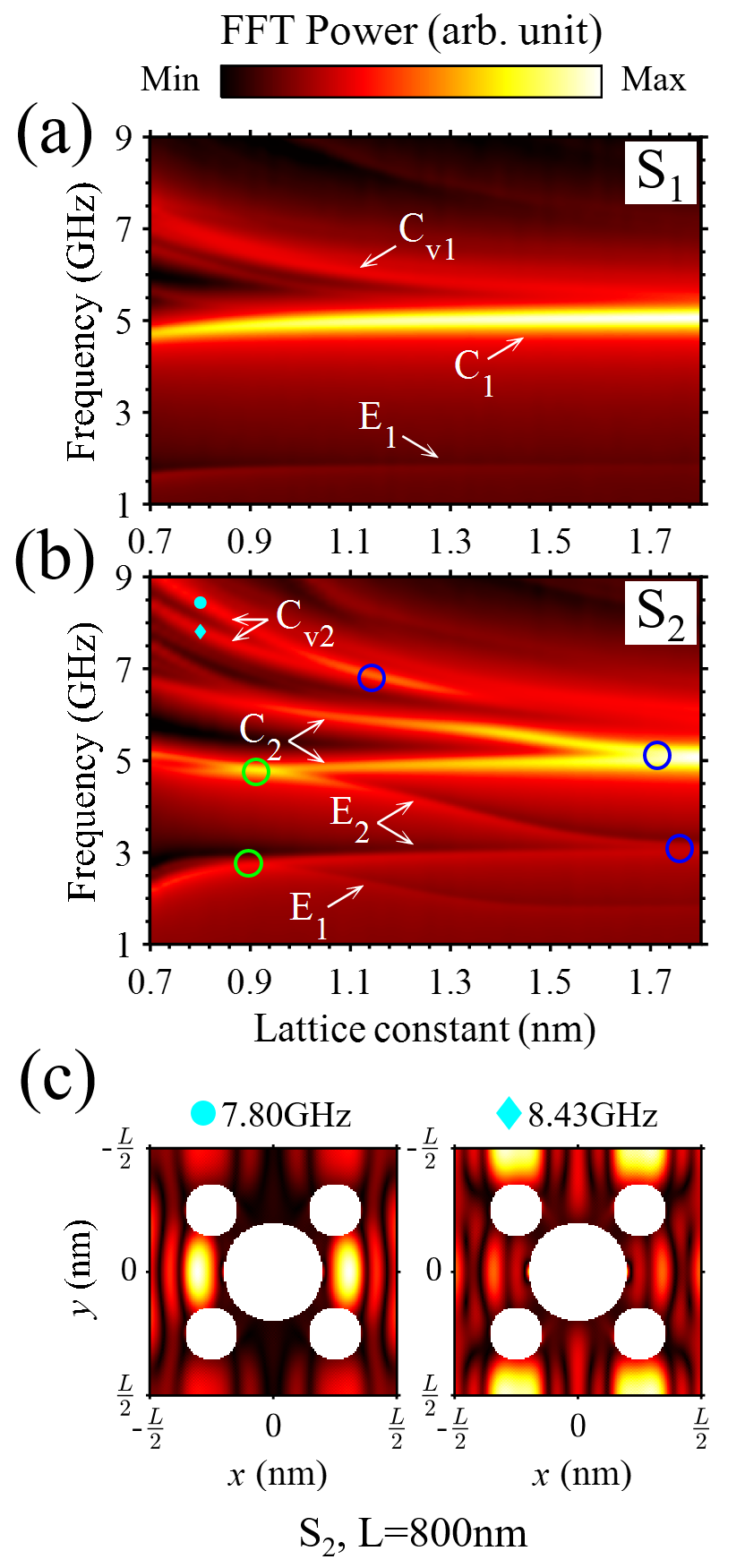


FIG. S5. Spin-wave spectra of (a) *S*_1_ and (b) *S*_2_ fractals for different lattice constants (*L* = 700 ~ 1800 nm) for fixed conditions of *D* = 300 nm and *H* = 30 mT applied in the +*x*-direction. (c) Spatial distributions of the FFT power for the *C_V_*_2_ doublet in *S*_2_ fractal of *L* = 800 nm. The split modes were reunited in a long channel width (blue circles). Two different modes (e.g. higher E_2_ and lower C_2_ in S_2_) crossed over in a short channel width (green circles).
