# Supplementary material for: Recursive evolution of spin-wave multiplets in magnonic crystals of antidot-lattice fractals
Source: Sci Rep. 2021 Nov 19;11:22604. doi: 10.1038/s41598-021-00417-0 (PMC8604906; doi:10.1038/s41598-021-00417-0)
Supplement: Supplementary file 2 — Supplementary Movies. [file 41598_2021_417_MOESM2_ESM.zip › Descriptions.docx]

**[SUPPLEMENTARY]**

**Recursive evolution of spin-wave multiplets**

**in magnonic crystals of antidot-lattice fractals**

Gyuyoung Park, Jaehak Yang, and Sang-Koog Kim^a)^

*National Creative Research Initiative Center for Spin Dynamics and Spin-Wave Devices, Nanospinics Laboratory, Research Institute of Advanced Materials, Department of Materials Science and Engineering, Seoul National University, Seoul 151-744, Republic of Korea*

^a)^ Correspondence and requests for materials should be addressed to S.-K. K (sangkoog@snu.ac.kr).

**Supplementary files**: Animation movies of S_2_ antidot-lattice fractal’s dynamic eigenmodes at bias magnetic field of 30 mT along +x-direction: the doublet of edge mode (E_2_) – ‘E2(2.93 GHz)’ and ‘E2(3.65 GHz)’, and the doublet of center mode (C_2_) – ‘C2(4.94 GHz)’ and ‘C2 (5.61 GHz)’. Each movie describes the magnetization (M_z_) oscillation inside S_2_ motif at the corresponding mode’s frequency.
